# Nucleation and growth of gold nanoparticles in the presence of different surfactants. A dissipative particle dynamics study

**DOI:** 10.1038/s41598-022-18155-2

**Published:** 2022-08-17

**Authors:** Rosa Suárez-López, Víctor F. Puntes, Neus G. Bastús, Carmen Hervés, Carlos Jaime

**Affiliations:** 1 Departament de Química, Universitat Autònoma de Barcelona, Bellaterra (Cerdanyola del Vallès), 08193 Barcelona, Spain; 2 Institut Català de Nanociència i Nanotecnologia (ICN2), CSIC and BIST, Campus UAB, Bellaterra, 08193 Barcelona, Spain; 3 Vall d’Hebron Institut de Recerca (VHIR), 08035 Barcelona, Spain; 4 Institució Catalana de Recerca i Estudis Avançats (ICREA), P. Lluís Companys 23, 08010 Barcelona, Spain

**Keywords:** Colloids, Computational chemistry, Nanoparticles, Coarse-grained models

## Abstract

Nanoparticles (NPs) show promising applications in biomedicine, catalysis, and energy harvesting. This applicability relies on controlling the material’s features at the nanometer scale. Surfactants, a unique class of surface-active molecules, have a remarkable ability to tune NPs activity; provide specific functions, avoid their aggregation, and create stable colloidal solutions. Surfactants also control nanoparticles’ nucleation and growth processes by modifying nuclei solubility and surface energy. While nucleation seems independent from the surfactant, NP’s growth depends on it. NP`s size is influenced by the type of functional group (C, O, S or N), length of its C chain and NP to surfactant ratio. In this paper, gold nanoparticles (Au NPs) are taken as model systems to study how nucleation and growth processes are affected by the choice of surfactants by Dissipative Particle Dynamics (DPD) simulations. DPD has been mainly used for studying biochemical structures, like lipid bilayer models. However, the study of solid NPs, and their conjugates, needs the introduction of a new metallic component. To represent the collective phenomena of these large systems, their degrees of freedom are reduced by Coarse-Grained (CG) models. DPD behaved as a powerful tool for studying complex systems and shedding some light on some experimental observations, otherwise difficult to explain.

## Introduction

Metal nanoparticles, especially gold nanoparticles (Au NPs) have attracted considerable attention due to their interesting physicochemical properties that differ from bulk materials. These unique properties, including biocompatibility and low cytotoxicity, make them valuable for biomedical applications, such as biosensing, bioimaging, cancer therapy, and drug delivery^1^. Moreover, their versatile surface chemistry allows functionalizing them with specific biomolecules, such as peptides, antibodies, antigens, and oligonucleotides^2^. This process provides biological specificity and compatibility to the NPs, improving their capability in drug loading and controlled release, and/or interacting with cell receptors ligands to get a definite targeting^3^.

The versatile surface chemistry that allows their easy functionalization is responsible for colloidal instability in the complex biological media where they are dispersed to be used^4,5^. NPs are very reactive due to the high surface-volume ratio, and tend to precipitate to minimize the surface tension in the colloidal suspension. Therefore, to ensure their colloidal stability, NPs are coated with organic molecules -surfactants- which provide electrostatic, steric or a mixture of both stabilization to NP’s surface^6–8^. They are present in the synthetic mixture from which NPs are produced and play a key role in regulating nucleation and growth processes. In addition, these same surfactants provide chemical functionality to the NPs or can be exchanged after synthesis once their nucleation and growth control have been completed.

In the case of Au, the general synthetic pathway for preparing monodisperse colloids involves the reduction of Au(III) to Au(0) in the presence of a surfactant to initiate the nucleation^9^. As soon as the concentration of Au(0) exceeds the solubility (saturation) limit, the nucleation starts being observed, and it increases until Au(0) concentration decreases below a critical supersaturation threshold^10,11^. In these conditions, the control of Au NP morphology can be achieved by adjusting the chemical nature of the surfactant (strength of their interaction with gold, surfactant chain length and functional group nature, among others). The most common surfactants are carboxylic acids, amines and thiols. The surfactant-to-gold binding strength follows, according to Pearson’s HSAB concept, the order C < O < N < S^12^. The use of sodium citrate (SC) as a reducing agent and stabilizer for the synthesis of Au NPs developed by Turkevich in 1951^13^ is still one of the most popular approaches. Brust and Schiffrin reported a two-phase synthetic strategy utilizing thiol-gold interactions to protect Au NPs with thiol-containing organic compounds, which can self-assemble onto the gold surface obtaining Au NPs with extremely narrow size distributions^14^. Likewise, amine-capped Au NPs were reported using primary amines for Leff et al. with similar results^15^.

The production of AuNPs has reached industrialization and there are many reports on their synthesis in different media and conditions. However, the control of monodispersity, colloidal stability and reproducibility of AuNPs are still a challenge^16^. Therefore, gaining insights into the processes of Au NPs formation, and the effects of surfactants in the nucleation and NPs growth are needed.

Computational methods provide new tools for understanding these systems, and exploring the mechanisms involved in the design and behaviour of conjugated NPs^17,18^. A wide variety of computational methods are available to researchers, going from ab initio to molecular mechanics. However, these methods usually work with all-atom models, and the study of complex systems is out of scale due to the many degrees of freedom to handle^19^. Alternatively, Coarse-grained (CG) models were developed to overcome this drawback, representing an attractive alternative to atomistic models. In CG simulations, sets of atoms are grouped in one *bead* allowing simulations to be run on much larger systems and on longer time scales^20–22^. To explain the collective phenomena or dynamics of these big systems, a new stochastic mesoscale method known as Dissipative Particle Dynamics (DPD) was developed. DPD combines CG models with Molecular Dynamics (MD) and Monte Carlo (MC) algorithm^23,24^. It has been used to study, among others, soft matter^25^, complex fluids^26^, lipid bilayers^27,28^, polymers^29^, proteins^30,31^ and colloidal systems^32^. Our group has been working with DPD to study the properties of different colloidal systems^33–36^ and lipid bilayers^37^.

In this work different syntheses of Au NPs have been studied by combining an atomistic model (DFT) with DPD. The studied surfactants were five: one of type O, two of type S, with different lengths, one of type N, and one of type C (linear hydrocarbon chain) in aqueous solutions. Although no experimental results using pure hydrocarbon chain surfactants exist, we based our work on Esumi et al*.* studies^38^. They reported that the particle size depends on the alkyl chain length and structure. All compounds were coarse-grained into beads (building-blocks). Gold beads behave similarly to gold atoms. Therefore, the aggrupation of gold beads is defined as clusters (also called as nuclei^39^) and clusters are grouped forming NPs. Results show the relationship between concentration of gold atoms and the size and the number of resulting NPs. Furthermore, the gold-to-surfactant ratio, as well as the nature, the chain length and the hydrophilicity of surfactants control the size and shape of the NPs.

## Results and discussion

**Figure 1:**
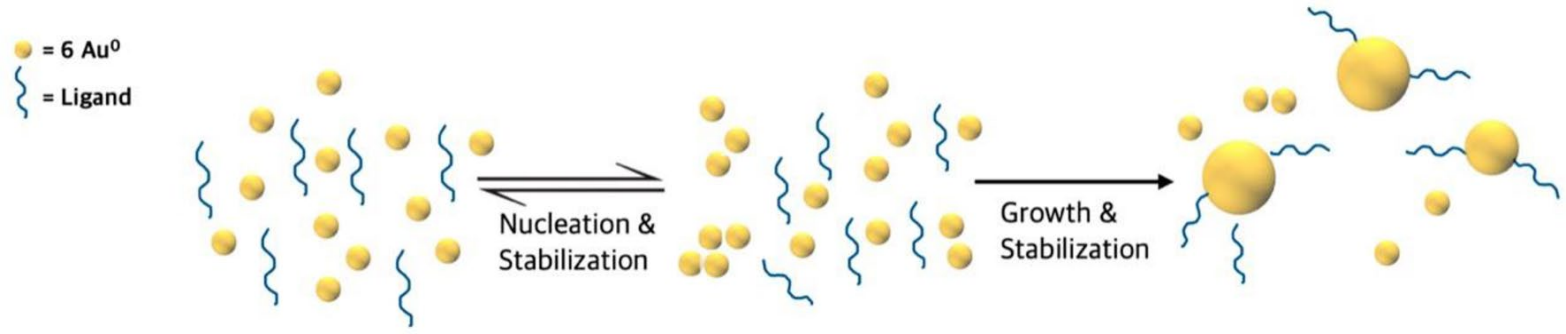
Schematic illustration of the general mechanistic steps in NPs synthesis.

This work uses DPD simulations to give insights into experimental results on NP synthesis that cannot be easily observed. It has been described that size control is achieved when the nucleation and growth processes are separated in time (Fig. 1)^40^. Nucleation is when a discrete particle of a new phase, a cluster, is formed in a previously single-phase system. These clusters are called nuclei in wet chemistry NP synthesis. These clusters have a critical radius that corresponds to the minimum size at which a particle can stay in solution without being redissolved. The particle’s free energy should be that critical for obtaining stable particles within a solution. The nucleation rate depends on the number of growing units and their mobility. In the growth stage, additional material is deposited on that cluster increasing its size and becoming an NP. This is controlled by the diffusion of growing species and by surface processes. In our work, a cluster is formed when roughly 3 gold beads (18 atoms of gold) join.

### Formation of gold clusters (nucleation step)

In this section, attention is centered on how gold and surfactant concentrations, and surfactant type influence the rate of clusters formation. The creation of gold clusters starts to be observed after reducing gold atoms (Au(III) to Au(0)). Thus, our gold beads were considered to be in their elementary state, i.e., once tetrachloroauric(III) acid (HAuCl_4_) is reduced by sodium citrate (SC) to Au(0). The working temperature was equal to 343 K. Full details of the simulations are shown in the Computational methods section**.**

As commented above, the nucleation rate depends on the number of gold units and their mobility. A critical radius and energy barrier must be surpassed to form a stable cluster^41^. The formation of these NPs involves two different physicochemical processes. The first process is needed to form stable clusters, while the second is necessary to grow them to obtain the final NPs.

Gold beads start to group from the beginning of simulations; however those aggrupations smaller than the critical nucleation radii redissolve indicating their instability. Stable clusters are usually formed after 945 ps. Clusters growth depends on gold concentration. At lower gold concentrations, clusters group slowly and the total number of clusters in the solution is high. On the contrary, using higher gold concentration, clusters group faster, growing larger, while the total number of clusters in solution accordingly decreases.

**Figure 2:**
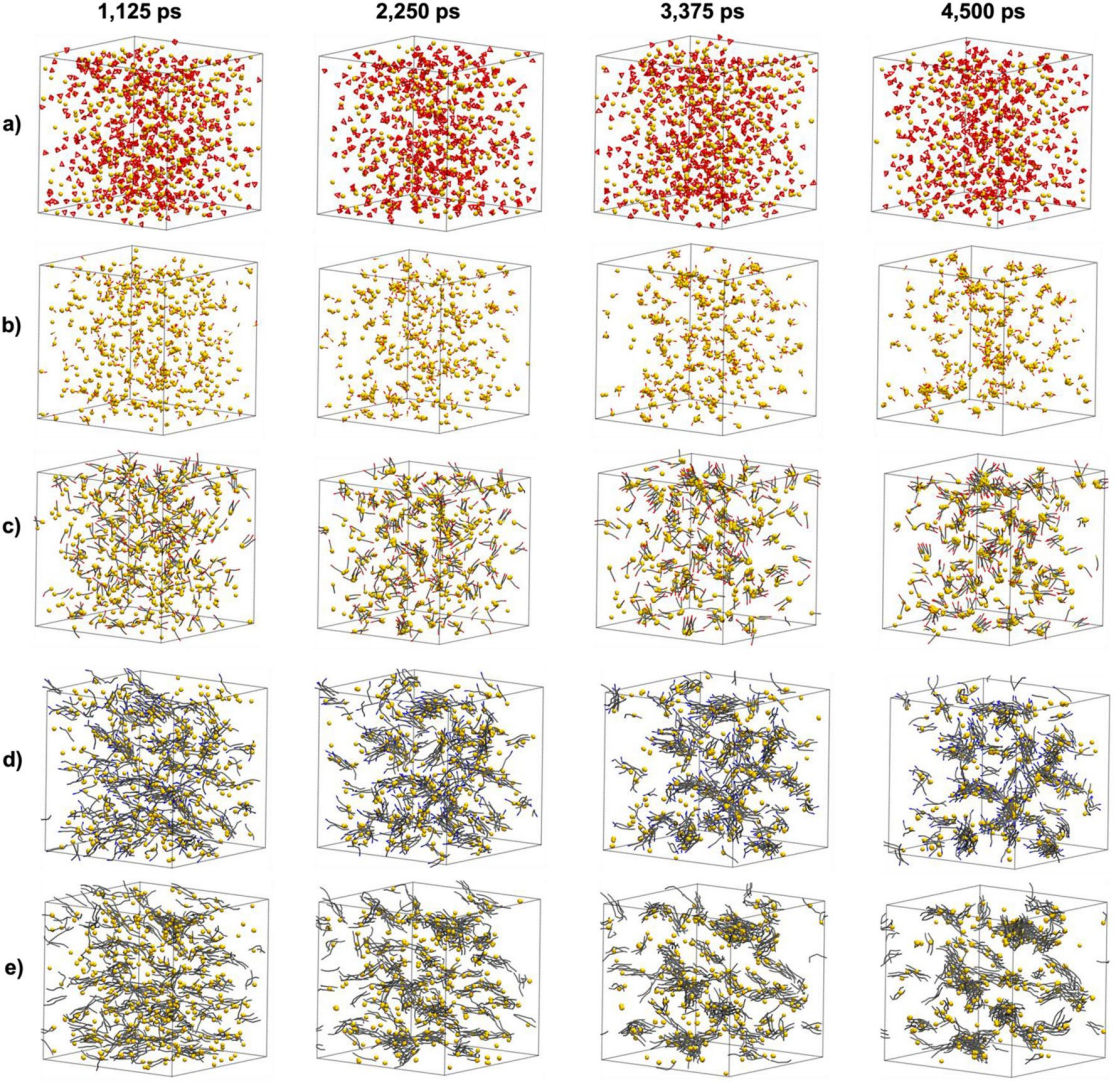
Snapshots taken at 1125, 2250, 3375 and 4500 ps. (**a**) O-type/Au. (**b**) S-short/Au. (**c**) S-long/Au. (**d**) N-type/Au. (**e**) C-type/Au. Gold beads were represented in CPK of drawing method, while surfactants were drawn with Licore method. Waters were removed and all surfactant bond radius were reduced for clarity.

Interestingly, simulations show how the type of surfactant determines nucleation. Main differences are based on surfactant structure, chemical nature and length. Results indicate that gold beads join faster when surfactants concentration is low, and when they have a poor affinity for gold. Clusters are formed fast when surfactants are also aggregated quickly, due to the hydrophobic effect (like MUA or C-type surfactants). Additionally, the formation of clusters is more difficult when using citrate molecules as surfactants. Citrate surfactants show a strong chelate effect (three anchoring sites) and their good interaction with water prevents the formation of small gold clusters. Five examples of simulations using 2/1 (Au/surfactant) molar ratios are shown in Fig. 2.

### Syntheses of Au NPs (growth step)

This study starts after gold ions are reduced; therefore, the reducing agent is irrelevant. The growth step is based on “bottom-up” approach implying the spontaneous agglomeration of gold beads. The interaction between surfactants and Au NP surface depends mainly on the functional group binding to the NP (in principle, following Pearson’s HSAB theory)^12^, together with the length and nature of the surfactant chain and its ω-functional group. All these factors influence the stability and reactivity of Au NPs. Our attention was centered in three well-known experimental procedures, which use O-, N- or S-type surfactants in water as solvent. Moreover, the behaviour of Au NPs in the presence of a new surfactant (C-type) in an aqueous solution was explored. Throughout the study, the surfactant concentration and the working temperature were varied. The conditions generating the different studied Au NP synthesis processes working at 343 K are shown in Table 1.

**Table 1:** Conditions generating the Au NP synthesis processes studied in this work at 343 K by varyingthe type of surfactants, and surfactant/Au ratio.

Surfactant type	Surfactant/Au ratio										
	0.06	0.2	0.33	0.6	1.0	1.2	2.6	3.0	3.2	5.0	5.4
S-short		1			3		4			10	
S-long		2			3		5			17	
O						3			2		3
N	2		3	3				15			
C	3		2	5				12			

S-short type is referred to mercaptopropionic acid (MPA) and S-long type to 11-mercaptoundecanoic acid (MUA).

Simulations containing O-, N- and S-type surfactants are inspired by Turkevich method^13^, Aslam et al.^42^ and Yonezawa and Kunitake’s experiments^43^, respectively. The first was based on mixing HAuCl_4_ with SC as a reducing and capping agent for Au NPs. Similarly, the second demonstrates that Au NPs could also be prepared in water directly by complexation with alkylamine molecules acting as a surfactant and reducing agent. In addition, primary amines containing long alkyl chains such as the ones employed here, are well-known to self-assemble and have been used as templating agents to synthesize other NPs. Finally, the third one, proposes preparative methods for obtaining stable dispersions of Au NPs using small water-soluble mercapto surfactants.

**Figure 3:**
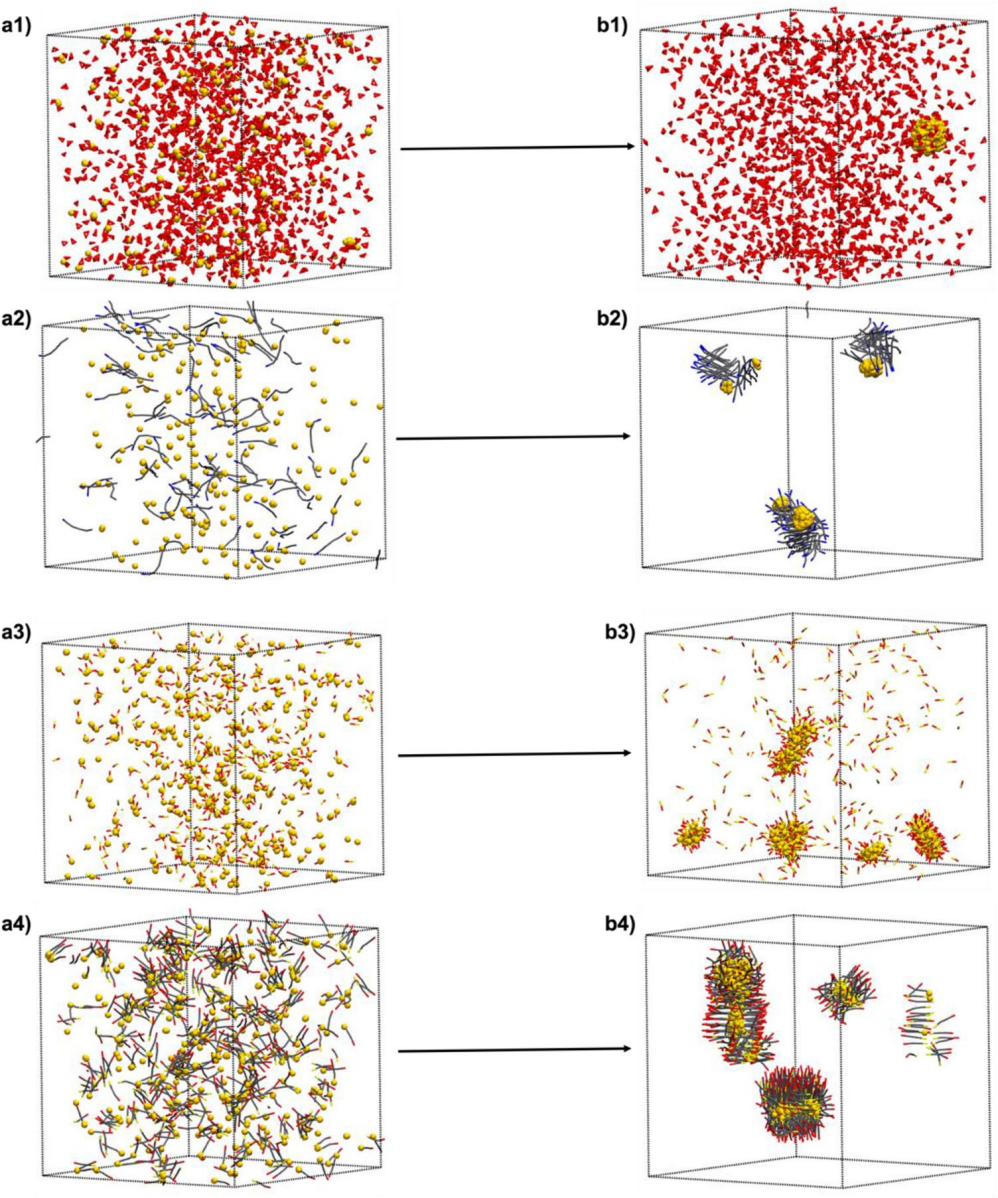
The initial point for all simulations is observed on the left (from **a1** to **a4**), and the final structures obtained after 1.35 μs are shown on the right (from **b1** to **b4**). (**a1**,**b1)** contain 500 golds beads mixed with 1350 citrate molecules at higher working temperature (373 K). (**a2**,**b2**) are obtained when 333 gold beads were mixed with 110 N-type surfactants. (**a3–b3)** and (**a4–b4)** contain 500 gold beads and 650 S-type surfactants (1/2.6 molar ratios). MPA molecules (**a3–b3**) are formed by two different beads (O-type and S-type), while MUA molecules (**a4–b4**) contain four beads. The lengthening of the surfactant can cause different effects such as the deformation of Au NPs. Note that hydrocarbon chains want to stay closer for minimizing their interaction with solvent. Gold beads and citrate molecules are represented by CPK drawing, while N-type and S-type molecules are represented using Licore drawing method. Water molecules are removed for clarity.

Citrate was chosen as a model for O-type surfactants, oleylamine for N-type surfactants , and MPA and MUA for S-type surfactants. All components were randomly distributed in a cubic box of 30 × 30 × 30 *d*_0_^3^ containing 800,000 water beads. Different surfactant to Au molar ratios (surfactant/Au) from 0.06 to 5.4 were used according to each synthesis. All procedures were done working at two different temperatures: 343 K and 373 K, and along 1.35 $\upmu$s. The employed soft repulsion parameters (SPR) are described in the Experimental section. Examples of the initial and the final point obtained when doing these simulations are illustrated in Fig. 3.

The main differences observed when applying different surfactants are explained by five unrelated parameters: gold-surfactant interaction, surfactant-solvent affinity, surfactants’ length, surfactant concentration, and temperature. First, the interaction gold-to-surfactant is different, and SRPs account for them (see “Experimental” section). Citrates are labile surfactants interacting weakly with gold beads by electrostatic stabilization (SRP Au/O = 18). Oleylamines are organic compounds that can interact with gold using the terminal N-type bead (SRP Au/N = 15) and contain hydrocarbon chains providing steric stabilization. Moreover, S-type surfactants interact strongly with gold beads (SRP Au/S = 5) associated with forming a pseudocovalent bond between them. Second, the surfactant-solvent affinity also affects the results. Several studies showed that the surface-solvent interactions affect considerably either the morphology or the size of NPs^44^. Due to its composition, O-type and MPA surfactants do not collapse onto Au NP surface. Citrates, and in some sense also MPA, molecules are formed by highly hydrophilic groups that are stable in water solutions due to their interaction with water molecules by hydrogen bonds^45^. In addition, computational studies showed that carboxylic acids headgroups of self-assembled monolayers can form hydrogen bonds with water molecules^46^. On the contrary, N-type and MUA surfactants have the tendency to get closer once attached to the Au NP surface to minimize as much as possible its interaction with water. This hydrophobic effect is caused when non-polar solutes are surrounded by polar solvents like water^47^. Accordingly, slightly different behavior can be observed in the disposition of these surfactants. In the case of N-type surfactants, the Au NPs are exposed to the solvent, while MUA molecules encapsulate gold beads forming isolated Au NPs. Third, regarding the surfactants carbon chain, MUA and oleylamine contain a hydrocarbon chain, which have the tendency to aggregate to avoid its interaction with water beads (solvent). Kaur et al. cloncluded that the hydrocarbon chain of Gemini surfactants plays an important role on NP–NP interactions^48^. The higher is the hydrocarbon chain the more hindrance between gold beads aggregation. Consequently, the growth of Au NPs is difficulted and the number of Au NPs is increased.

Fourth, the surfactant concentration modulates the size of Au NPs because by increasing it, a higher number of Au NPs are formed. At high concentrations the encounter between gold beads is laborious due to the hindrance of surfactants and to a favoured gold-surfactant interaction. Simulations show that, for instance, using 0.2 molar ratio a unique NP is obtained, while using 5.0, the total number of Au NPs increased to 17. Moreover, these simulations corroborate the cooperative growth of molecular domains onto the NP surface until surface saturation. As a consequence, at higher concentrations of surfactants, unidimensional growth is promoted, recalling CTAB-Gold nanorod mechanisms of growth^49^.

**Figure 4:**
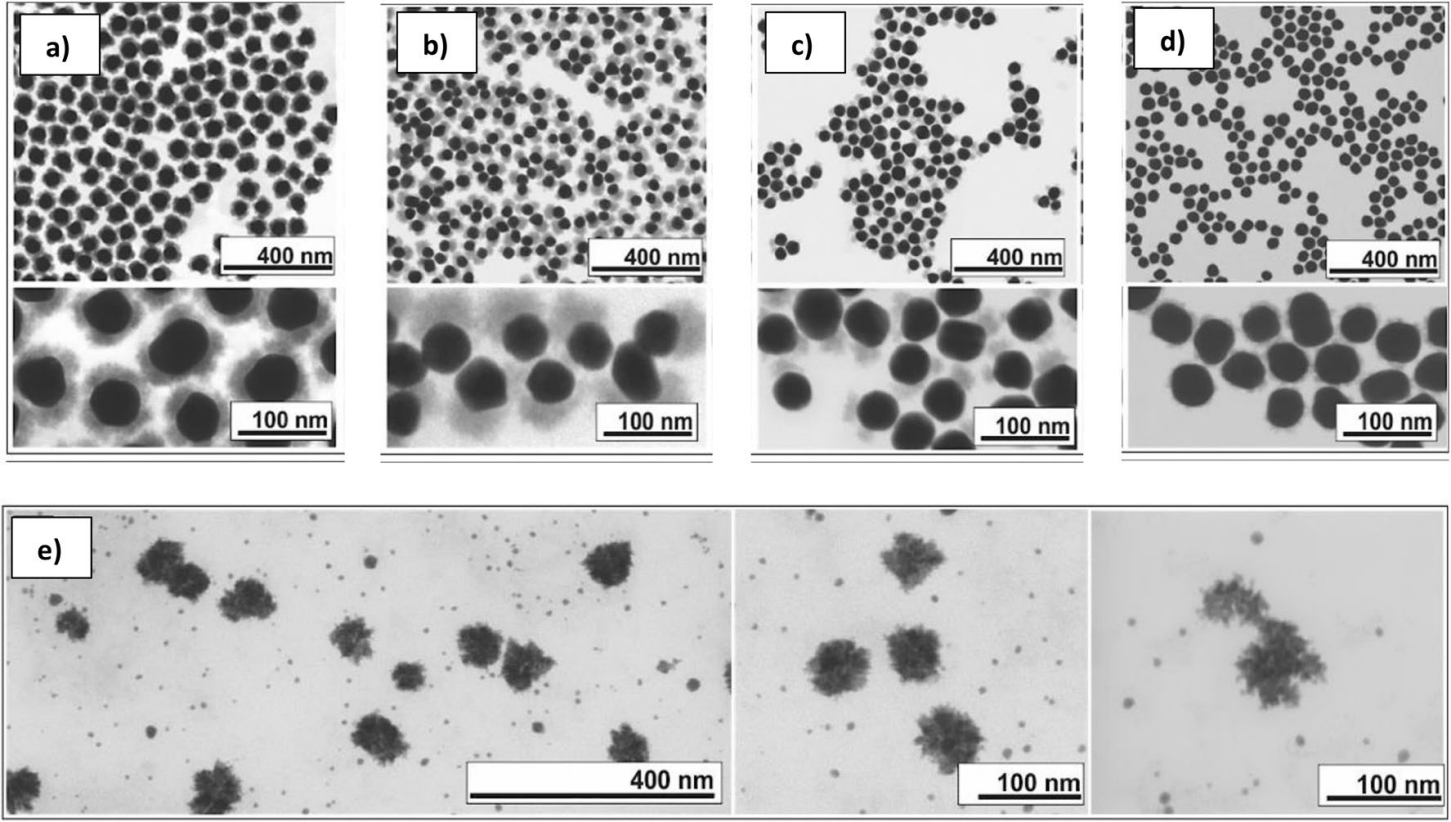
TEM images of Au NCs at different MUA molecules to Au surface ratios. (**a**) 0, (**b**) 0.5, (**c**) 50, (**d**) 500 and (**e**) 5000.

To further verify these premises, we performed experiments for Au NP synthesis in the presence of both a defect and an excess of MUA. In Fig. 4, citrate-stabilized Au NPs of 50 nm in diameter were first synthesized^50^ in a deficiency of MUA, at concentrations where there is no enough MUA to cover all the NPs surface completely. These Au NPs were used as seeds for the growth of a CeO_2_ coating^51^. As expected, while a homogeneous CeO_2_ layer is grown in the absence of MUA, the formation of separated domains in the form of heterodimer-like structures is observed as MUA concentration is increased (Fig. 4c). The selective deposition of CeO_2_ can be explained by the formation of MUA domains that protect certain areas of the NP surface. As a result, the surface available exposed is reduced, thereby generating binary Au-CeO_2_ structures. Otherwise, if subsaturation MUA was evenly distributed on the surface, the corona would take longer and make less dense coronas, but isotropic. Accordingly, the architecture of the final NPs is determined by the concentration of MUA used in the experiment, comprising also complete core–shell (Fig. 4a) and clover-like structures (Fig. 4b). Finally, when the concentration of MUA is the highest tested, the growth of CeO_2_ is almost suppressed (Fig. 4d). For the same reasons, the spherical symmetry of the Au NPs is lost when the synthesis of Au NPs is performed in the presence of a high concentration of MUA. Furthermore, the presence of MUA promoted the substantial decrease of Au NP surface energy and the template-assisted growth of Au into spike structures confined in elongated MUA micelles (Fig. 4e).

Finally, when the simulated temperature is increased to 373 K, the rate of synthesis is significantly accelerated increasing the collision rate between gold beads which is ultimately translated into larger Au NPs. Figure 3a1, b1 shows the initial and the final point when 1350 citrate molecules are mixed with 500 gold beads at 373 K. The final structure is composed by a unique Au NP containing all gold beads. Results obtained at 373 K. with N- and S-type surfactants can be observed in the Supplementary Information.

### C-type surfactants in aqueous solution

Octadecane was the hydrocarbon chain selected for representing a C-type surfactant. All jobs contain 333 gold beads and different number of C-type surfactants arbitrary distributed to make 0.06, 0.33, 0.6 and 3 surfactant/Au molar ratios. The procedures were performed at 70 and 100 °C. SRPs of C-type are defined in the 6th column of the inset in the Experimental section. Considering C-type molecules as hydrophobic compounds, SRPs between gold and those surfactants are higher than the others (SRP Au/C-type = 55). Thus, we expect a poor interaction between these compounds with gold.

**Figure 5:**
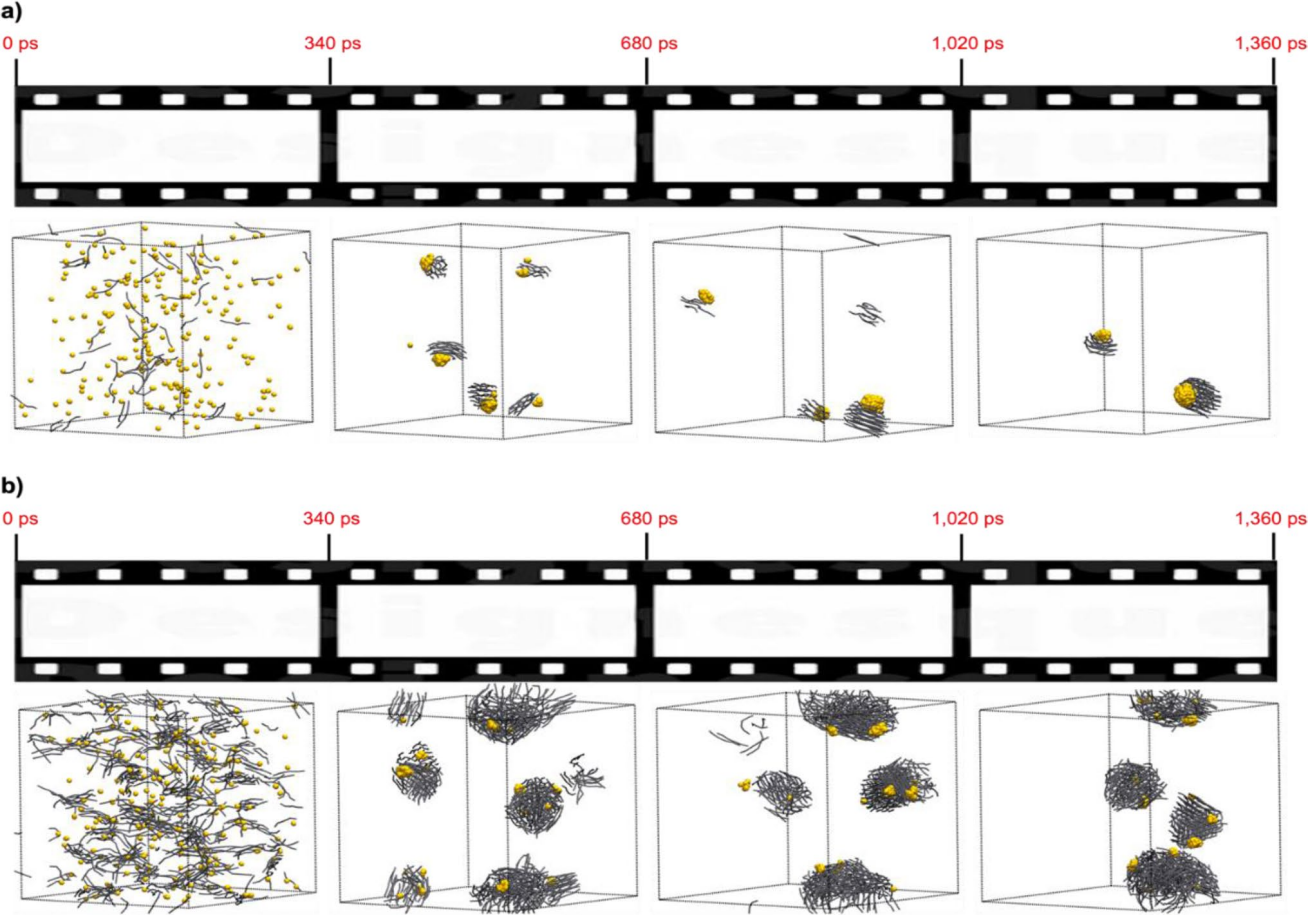
(**a**) 333 gold beads are mixed with 55 C-type surfactants. (**b**) 333 gold beads mixed with 500 C-type molecules. Water molecules are removed for clarity. All compounds are randomly distributed in a cubic box of 30 × 30 × 30 *d*_0_^3^*.* Gold beads are represented using CPK model of VMD while C-type molecules are shown in Licore representation.

Results show that, despite the almost null affinity of octadecane molecules for gold, C-type surfactants end up on Au NP surface because they are expulsed from water. At higher concentrations of C-type, the mobility of gold beads is reduced. In addition, an increase in temperature weakens the gold-surfactant interactions and its protective effect. Thus, the number of Au NPs is decreased at the expense of their increase in size. Simulations show that, hydrocarbon chains are agglomerated, and Au NPs are exposed to the solvent. The main reasons can be related to the hydrophobicity of C-type surfactants^47^. Their poor interaction with water solvent (SRP W/C = 82) enhances their aggregation. An example of our simulation is shown in Fig. 5. Surfactants get closer to minimize their interaction with water and they are assembled onto the Au NPs surface (SRP Au/C = 55). As a result, C-type surfactants started to attach to Au NPs, leaving a part of them exposed to the solvent. If two Au NPs were sticked to the same group of surfactants their possibility of getting together decreases. Thus, the number of Au NPs is increased. Moreover, we can see that the higher the number of surfactant molecules, the more Au NPs are obtained, considering that temperature (343 K) and box dimensions are kept constant.

Our simulations show that the formation of stable clusters can be observed when the concentration of surfactants is low enough. As experimentally observed, in excess of strongly binding surfactant, the nucleation and growth process are slowed until virtually stopped. The formation rate and size of the Au clusters depends on the chemical nature and the length of the surfactants. Kinetically speaking, the velocity of gold beads to come together is related to their concentration, as far as it is above supersaturation. Stable NPs can be observed when stable clusters join. The growth of these clusters can be tuned by the surfactants taking part in the synthesis. Obeying Pearson’s HSAB law^12^, we can conclude that generally, all surfactants that interact weakly with gold, such as citrate molecules, produce larger NPs. On the other hand, those that interact strongly (thiol groups for instance) yield smaller and more numerous Au NPs. Furthermore, the length and the structure of surfactants should also be considered. The number of Au NPs can increase faster when surfactants are shorter due to the lack of steric hindrance that forbid the encounter between gold beads. Moreover, if we compare two surfactants with similar structure but containing different terminal groups, their interaction with the solvent also affect the results. Simulations also verify that according to CTAB-Gold nanorod mechanisms of growth^49^, higher concentrations of surfactants give more unidimensional Au NPs growth.

## Conclusions

A study of Au NPs synthesis is presented using DPD method for being capable of explaining experimental results, although introducing a new metallic nature to a computational method created for studying biochemical systems could be difficult. Herein, we have presented the possibility of studying nanochemical approaches from the nucleation state to the growth step. This study indicates that computational studies are useful to illustrate a wide range of applications in the field of biomedicine and energy harvesting materials. In addition, we can conclude that DPD is behaving as a powerful tool for studying complex systems in reasonable periods of time, despite its limitation of losing atomistic details.

## Methods

### Computational methods

#### Mesomolecular (CG) model

**Figure 6:**
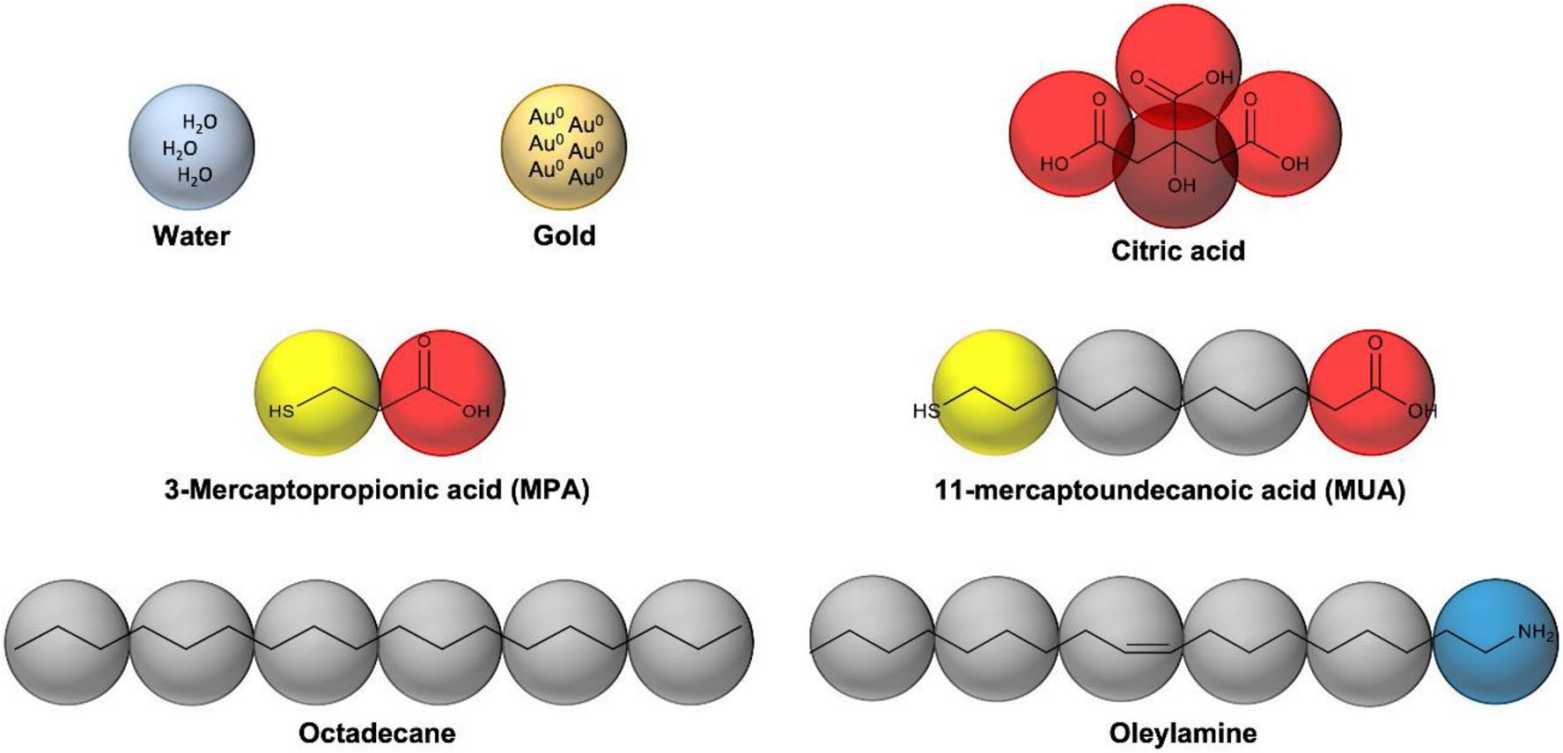
Mesomolecular model: the coarse-grained applied for all components is represented with different types of beads. Water (W-type) and gold beads (Au-type) are depicted in light blue and yellow, respectively. Both contain three gold atoms or water molecules. Water as solvent and gold as AuNP are formed by large number of individual beads. Five kinds of surfactants with different length and bead types are used. Citrate anion is formed by two different beads, three terminal anion-like fragments (O-type) and a central alcohol group (OH-type), depicted in light and dark red, respectively. Both MPA (S-type short) and MUA (S-type long) contain a terminal thiol group depicted in light yellow (S-type), and a hydrophilic anion fragment (O-type), depicted in red. Furthermore, MUA’s chain needs to use hydrocarbon beads (C-type), depicted in light grey. Oleylamine also contain two different types of beads, a head group formed by an amine group (N-type), depicted in dark blue, and five tail beads of C-type (hydrocarbon beads). The octadecane is conformed by six equal beads of C-type.

Usually, one *bead* is formed by three or four “heavy” atoms (non-hydrogens) together with all the hydrogens linked to them, although there are papers where much larger beads are formed^52^. In our model, one *bead* comprises three heavy atoms, except for gold. All beads must have the same size and since a pure gold atom occupies half the volume of one water molecule, one gold bead is formed by six gold atoms. The studied systems always contained three components: water, gold and surfactant. Five different surfactants were considered: MPA, MUA, citrate anion, oleylamine and octadecane. Each bead should behave similarly as the set of atoms that contains; consequently, seven types of beads were used (see Fig. 6).

### DPD formulation

The original DPD formulation was modified to ensure the proper thermal equilibrium state and deriving hydrodynamic variables^53,54^^.^

The spatial–temporal evolution of each DPD particle is governed by Newton's equations of motion (Eq. 1):

1 $$\frac{d\overrightarrow{{r}_{i}}}{dt}={\overrightarrow{v}}_{i}\frac{d{\overrightarrow{v}}_{i}}{dt}=\overrightarrow{fi}$$

where, $\overrightarrow{{r}_{i}}$ is position of particle *i*, ${\overrightarrow{v}}_{i}$ is the velocity of particle *i*, and $\overrightarrow{{f}_{i}}$ is the total force acting on bead *i*. For simplicity, all masses are normalized to 1. The total force exerted on bead *i* contains three pairwise additive forces (Eq. 2):

2 $${f}_{i}={\sum }_{j\ne i}\left({F}_{ij}^{C} +{F}_{ij}^{D}+{F}_{ij}^{R}\right) $$

where ${F}_{ij}^{C}$ refers to a conservative force, ${F}_{ij}^{D}$ to a dissipative force and ${F}_{ij}^{R}$ to a random force. The conservative force represents the chemical behaviour of the bead^55,56^, and contains two contributions. The first one is defined through Eq. (3):

3 $${F}_{ij}^{C}={a}_{ij}{w}^{c}\left({r}_{ij}\right){\widehat{r}}_{ij}$$

where ${a}_{ij}$ is the soft repulsive parameter between particles *i* and particle *j*, $\widehat{{r}_{ij}}$ is a unit vector in the direction of ${r}_{ij}$, ${w}^{C}$ is a weight function, and ${r}_{ij}={r}_{i}-{r}_{j}$. It derives from a soft potential that tries to capture the effects of the pressure between different particles. The second refers to the elastic contributions, which derive from the system geometry and contains stretching and bending parameters (Eqs. 4 and 5):

4 $${F}_{s}=-{K}_{r}\left({r}_{ij}-{r}_{eq}\right){\widehat{r}}_{ij} $$

5 $${F}_{\theta }=-\nabla \left(\frac{1}{2}{K}_{\theta }{\left(\theta -{\theta }_{0}\right)}^{2}\right)$$

Equation $(4)$ describes the harmonic force used to tie two consecutive beads *i* and *j* in a chain, and Eq. (5) is the bond-bending force between consecutive bonds, to control the chain flexibility and strength (see Supporting Information for more details).

### Determination of DPD interacting parameters (${a}_{ij}$)

A set of interacting parameters ${a}_{ij}$’s between beads must be determined to simulate a system (see Eqs. (3) and (6))^57^.

6 $${F}_{ij}^{C}=\left\{\begin{array}{l}{a}_{ij}\left(1-\frac{{r}_{ij}}{{R}_{c}}\right)\widehat{{r}_{ij}}\left({r}_{ij}<{R}_{c}\right)\\ 0\left({r}_{ij}\ge {R}_{c}\right)\end{array}\right.$$

$${a}_{ij}$$’s are referred to *as DPD soft repulsive interaction parameters (SRP)* between particles *i* and *j* (they should be $${a}_{ij}>0$$)^58^. $$\widehat{{r}_{ij}}$$ is a unit vector in the direction of $${r}_{ij}$$, $${R}_{c}$$ is the cutoff radius (normally equal to 1) for the repulsive interaction, and $${r}_{ij}={|r}_{i}-{r}_{j}|$$^59^ is the distance between particles *i* and *j.* These interactions are set to mimic that hydrophobic entities repel water (high SRP value), while hydrophilic groups attract water (low SRP value). Therefore, SRPs arise from water-water interaction ($$\mathrm{W}/\mathrm{W}=25$$). Furthermore, SRP can be considered as an expression of the association constant between the chemical species represented by each bead type. Thus, SRPs should be exponentially related with the Δ*E*_*binding*_ obtained by Gaussian software package^60^ (Eq. 7$$)$$^61,62^.

7 $$\Delta {E}_{binding}={E}_{system}-\left({E}_{{component}_{1}}+{E}_{{component}_{2}}\right)$$

Geometry optimizations were carried out with Density functional theory (DFT) calculations using the Minnesota M06-2X functional^63^, and with LANL2DZ basis^64,65^. Polarizable continuum model of water using the integral equation formalism IEFPCM was used to simulate the effect of solvent (water)^66,67^.

**Figure 7:**
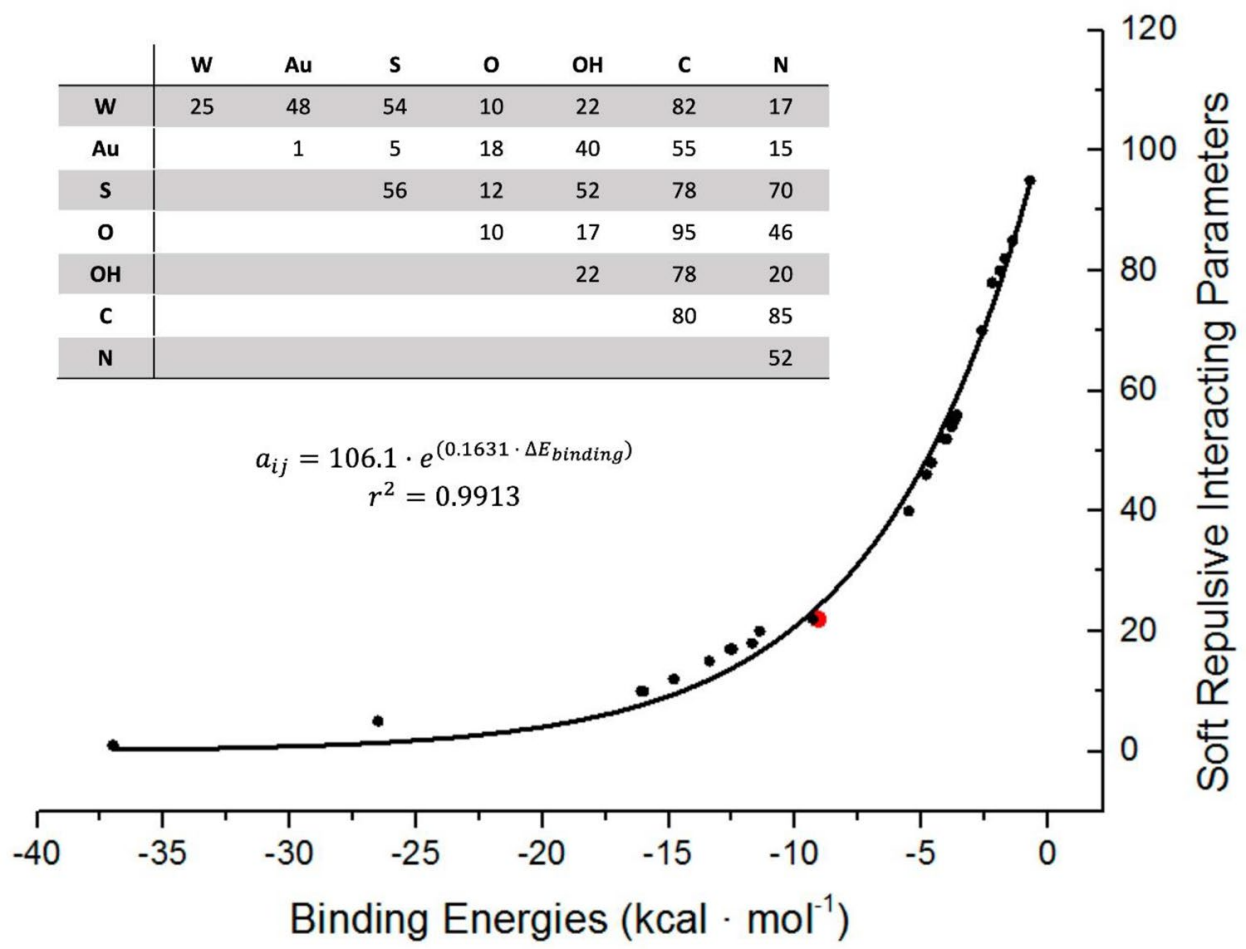
Exponential correlation plot between binding energies at LANDL2DZ/M062X level vs. DPD SRP. Red point represents water-water bead interaction (SRP W/W). Inset: Set of resultant SRP used in this work.

The resultant matrix of SRP values and the exponential function between SRPs and ΔE_bindings_ are depicted in Fig. 7 (more details on this item can be obtained from the Supporting Information).

These repulsive parameters followed the tendency of those used for studying lipid bilayers using the same DPD program^31,68^. However, cross terms (${a}_{ij}$) between gold and thiols were evaluated considering a thiolate for representing the strong gold-thiolate bond (${a}_{Au,S^{-}}$), considered as a pseudocovalent bond.

### Simulation conditions

All simulations were performed in an NPT ensemble using a dimensionless units system (reduced units, r.u.). For our parameterization, ρ = 3, m = 3, ${V}^{w}$= 30 Å, *d*_*0*_ = 6.47 Å, and the friction constant (λ) necessary for evaluating the dissipative force was set equal to 4.5. A time step of 0.03 τ was always used, and the simulation length was varied depending on the procedure followed. To observe the effect of temperature two different working temperatures were used: 0.42 and 1 r.u. (equivalent to 343 K and 373 K, respectively).

All simulations were performed in a cubic water box of ${L}_{x}\times {L}_{y}\times {L}_{z}=30\times 30\times 30{ d}_{0}^{3}$ and 240,000 water molecules were used in addition with variable, and randomly distributed, amounts of gold beads and surfactant molecules depending on surfactant/Au ratios. Simulation lengths were always equal to 1.35 μs.

Figures 2, 3, and 5 were obtained using VMD program^69^ rendered with the Tachyon option^70,71^.

## Experimental section

### Reagents

Tetrachloroauric(III) acid trihydrate (HAuCl_4_·3H_2_O, 99.9%), sodium citrate dihydrate (HOC(COONa)(CH_2_COONa)_2_ · 2H_2_O, ≥ 99%), cerium (III) nitrate hexahydrate (Ce(NO_3_)_3_ · 6 H_2_O, 99%) sodium hydroxide (NaOH, ≥ 99%), and 11-Mercaptoundecanoic acid (MUA, HS(CH_2_)_10_CO_2_H, 98%) were purchased from Sigma-Aldrich and used as received. All solutions were prepared in Milli-Q water.

### Synthesis of Au@CeO_2_ NCs

Citrate-stabilized Au NCs of 50 nm in diameter were synthesized from gold chloride and sodium citrate according to methods previously developed by our group^50^. The Au NCs were further functionalized with MUA and used without further purification as seeds for the CeO_2_ coating following a well-defined protocol developed by the group^51^. Ligand exchange was performed by adding known amounts of MUA molecules to Au NPs solutions under vigorous stirring. In detail, the ratio of MUA molecules to Au surface atoms was varied from 0, 25, 100 and 5000 which corresponds to the addition of 0, 0.025 mL, 0.25 mL, 2.5 mL, and 25 ml of MUA 1 mM to a volume of 12.5 ml Au NPs. The Au surface was calculated according to the size (50 nm) and concentration of Au NPs (6.04 10^10^ NP/mL, which corresponds to 7.6 10^14^ nm^2^/mL). The mixture was allowed to react for 12 h, when no further peak evolution was detected by UV–vis spectroscopy. MUA-functionalized Au NCs were purified and further used as a seeds for the growth of a CeO2 shell following a synthetic approach previously developed by our group^51^.

### Characterization techniques

Microscope analyses were carried out on an FEI Magellan 400L XHR SEM operating at 20 kV. Samples were centrifuged and dispersed in water previous to their deposition (10 μL) on an ultrathin formvar-coated 200-mesh copper grid (Ted-pella, Inc.). Average size and size distribution of the samples were measured using ImageJ software by counting at least 500 particles.

## Supplementary Information

Supplementary Information.

## Data Availability

Request for any data should be addressed to the corresponding author (C.J.).
