# Supplementary material for: Nucleation and growth of gold nanoparticles in the presence of different surfactants. A dissipative particle dynamics study
Source: Sci Rep. 2022 Aug 17;12:13926. doi: 10.1038/s41598-022-18155-2 (PMC9385746; doi:10.1038/s41598-022-18155-2)
Supplement: Supplementary file 1 — Supplementary Information. [file 41598_2022_18155_MOESM1_ESM.pdf]

Supplementary Information for:

Nucleation and Growth of Gold nanoparticles in the Presence of different surfactants. A Dissipative Particle Dynamics Study

Rosa Suárez-López,[1] Víctor F. Puntes,[2,3,4] Neus Bastús[2], Carmen Hervés[2] and Carlos Jaime[1,*]

[1]Department of Chemistry, Universitat Autònoma de Barcelona, 08193 Bellaterra (Cerdanyola del Vallès), Barcelona, Spain

[2]Institut Català de Nanociència i Nanotecnologia (ICN2), CSIC and BIST, Campus UAB, 08193 Bellaterra, Barcelona, Spain

[3]Vall d'Hebron Institut de Recerca (VHIR), Barcelona, 08035, Spain

[4]Institució Catalana de Recerca i Estudis Avançats (ICREA), P. Lluís Companys 23, Barcelona, 08010, Spain

**carlos.jaime@uab.cat*

Results and discussion

Formation of gold clusters (nucleation step)

The total number of clusters formed is measured using different Au/Surfactant molar ratios at the same working temperature (343 K). Water box is formed by 80,000 beads of water. Initially, all compounds are randomly distributed along the box. The real time in all simulations is equivalent to 4.5 ns.

Simulations show that the formation of clusters is favored when the number of gold beads is increased. Moreover, the structure and as well as, the length of surfactants play an important role on gold cluster formation. Thus, having a hydrocarbon chain on its structure causes faster aggregation between surfactants and for this reason, gold beads can join easily.

Syntheses of Au NPs (growth step).

O-type surfactant (Turkevich method). The components use in these experiments are shown in Fig. **S1**. Simulations always contain the same number of gold beads and different number of citrate molecules arbitrary distributed in a cubic box of 240,000 water molecules.

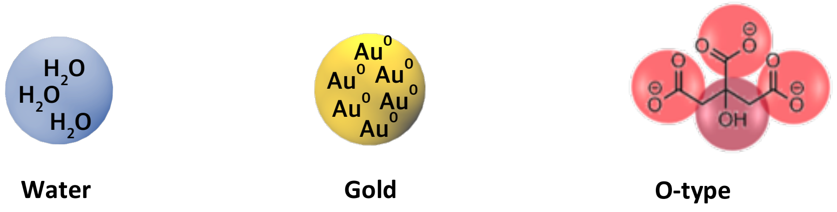

Figure S1- CG representation of solvent (water) depicted in light blue, gold in golden and citric acid depicted in red. Surfactants are formed by two hydrophilic beads; the ones that represent anion-like fragments surfactants (type O) and a central alcohol group (type OH).

Results using two different temperatures are seen in Fig. **S2 (a)** 343 K of working temperature. **(b)** 373 K of working temperature.

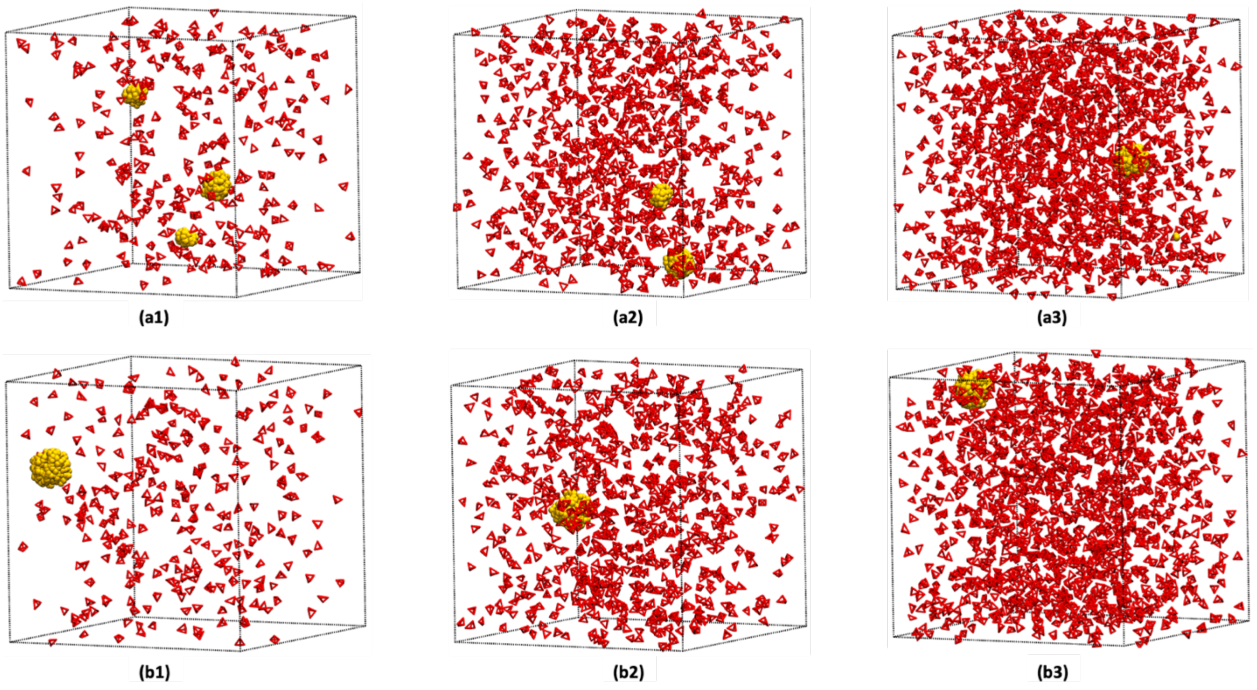

Figure S2- Resultant snapshots of the final structures (1.35 μs) when the number of citrates is changed. Au/Cit molar ratios are 1/0.6, 1/1.6 and 1/2.7 from the left to the right. Water molecules are removed for clarity.

The number of Au NPs varies depending on Au/surfactant molar ratios. An increasing of temperature gives a unique AuNP (**(b1)-(b3)**).

N-type surfactants (oleylamine protected Au NPs). The whole system is composed by three different components (see Fig. **S3**). All of them are randomly distributed in a water box composed by 240,000 water molecules.

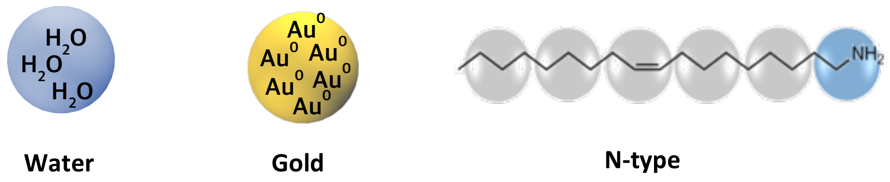

Figure S3- CG model applied for water (solvent), gold and oleylamine surfactants. Note that oleylamine surfactants are formed by two different types of beads: C-type bead (depicted in gray) for representing hydrocarbon chains and a terminal N-type bead (depicted in dark blue) which interacts strongly with gold beads (depicted in golden).

Final structures obtained when temperature is equal to 343 K (Fig. **S4 (a)**) and 373 K (Fig. **S4 (b)**) are shown below.

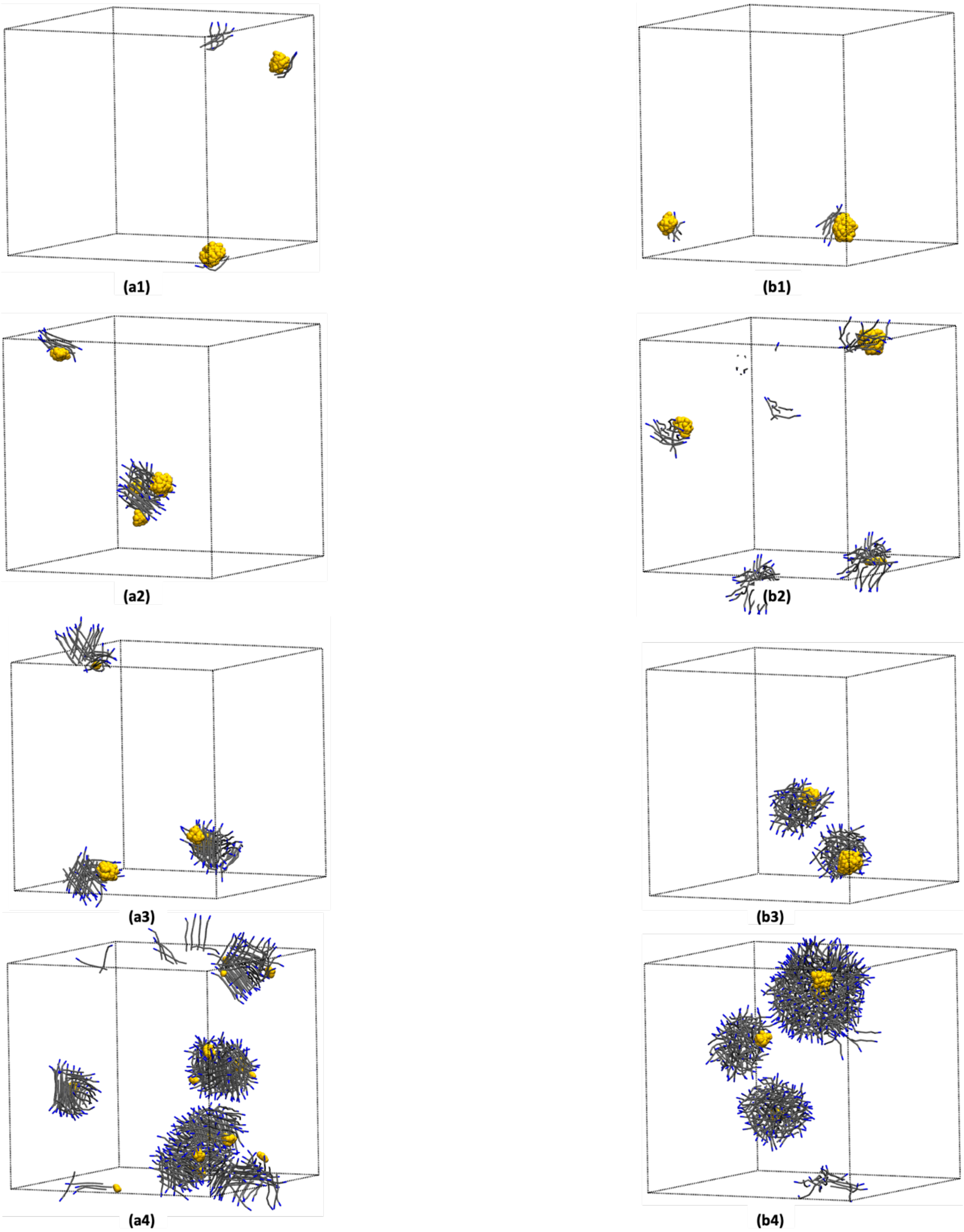

Figure S4- Ending structures (1.35 μs) of all jobs when using 343 K (from **(a1)** to **(a4)**) and 373 K (from **(b1)** to **(b4)**) of working temperatures. Water molecules are removed.

The number of Au NPs is affected when the temperature is increased. Fig. **S4** shows that an increment of oleylamine molecules gives bigger number of Au NPs.

S-type surfactants (anionic mercapto surfactant-stabilized Au NPs). Two different systems are created for studying the interaction between gold and S-type surfactants. The first one contains 240,000 water molecules (solvent), 500 gold beads (equivalent to 1,500 gold atoms) and different quantities of S-type short surfactants according to Au/surfactant ratios. The second one contains the same number of water molecules, gold beads and quantities of S-type long surfactants but they contain a larger hydrocarbon chain. CG model applied could be seen in Fig. **S5**.

The final snapshots get from all the simulations are shown in Fig. **S6** for S-type short surfactant and **S7** for S-type long surfactant. Note that in both cases, **(a)** pictures are obtained when using lower temperatures (343 K) and **(b)** when working with higher temperatures (373 K).

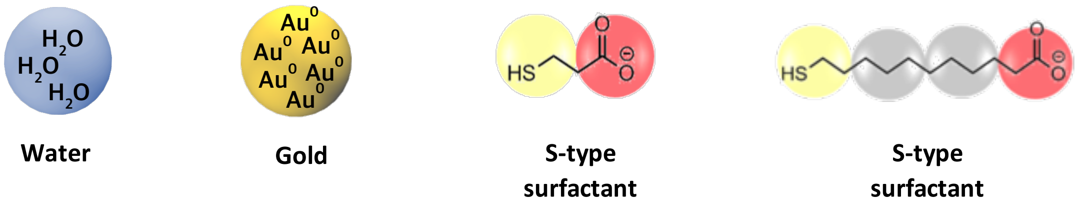

Figure S5- CG model applied for each component. Three water molecules are grouped to one bead (depicted in light blue) and represent the solvent box. Three gold atoms formed one golden bead. And, both S-type surfactants contain one S-type bead depicted in yellow and one O-type bead depicted in red. Moreover, the S-type long surfactant contain two C-type beads depicted in gray.

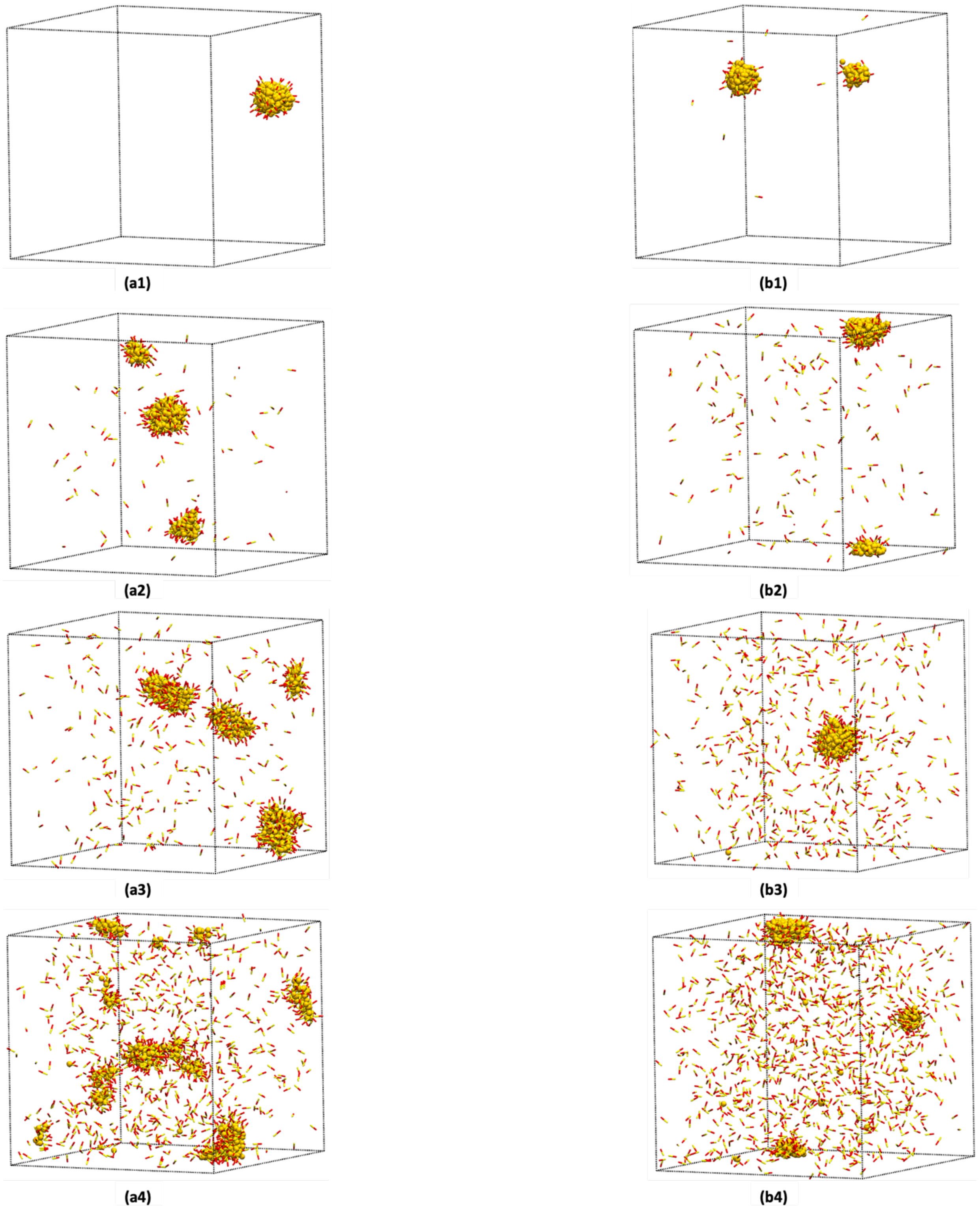

Figure S6- Ending structures obtained in 1.35 μs when temperature goes from 343 K **(a)** to 373 K **(b)**. water molecules were removed for clarity.

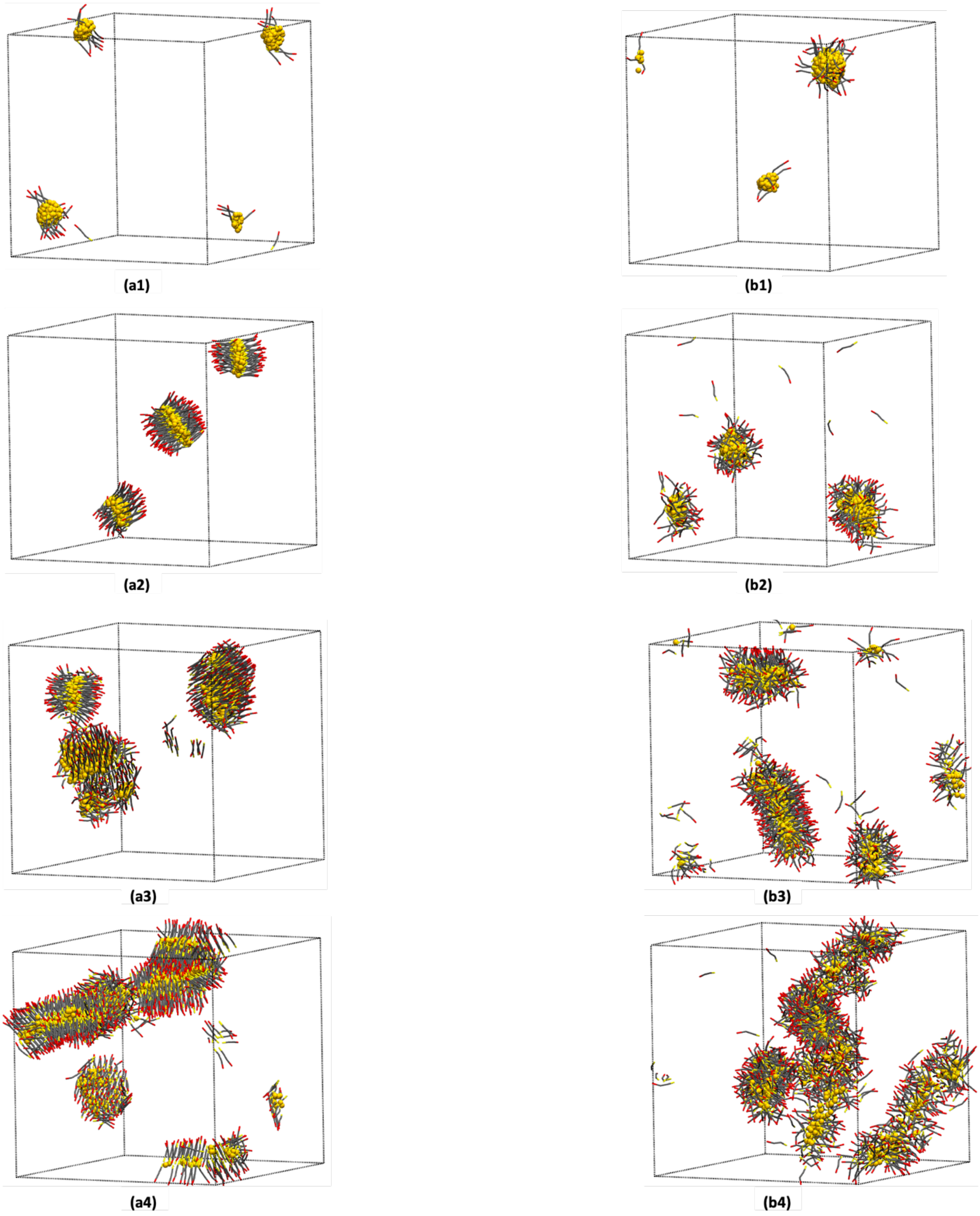

Figure S7- Ending structures obtained in 1.35 μs when temperature goes from 373 K **(a)** to 100 K **(b).** Water molecules were removed for clarity.

Results show that; on one hand, gold interacts strongly with S-type surfactants than the others. And, on the other hand, increasing the length of thiol groups leads to deform nanoparticles. Moreover, the fact of containing hydrocarbon chains cause the adsorption of surfactants to Au NPs surface for maximizing the C-type/C-type interaction and to avoid their interaction with water solvent molecules. Furthermore, the increment of temperature favors the faster movement of compounds and causes a decrease of Au NPs. Note that, when the molar ratio Au/S-type is higher the number of gold aggrupation is reduced giving misshape gold nanospheres.

C-type surfactant in aqueous solution. The growth is done when gold beads are mixed with octadecane molecules in a cubic box of 80,000 water molecules. Fig. **S8** shows the CG applied with all components.

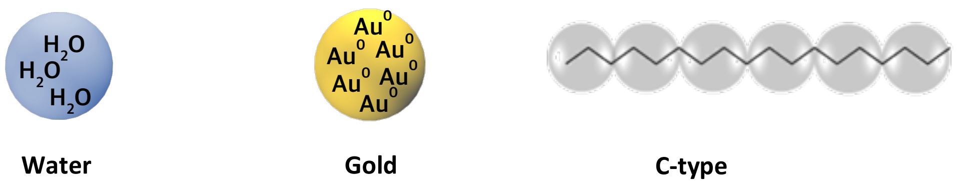

Figure S8- CG model of the different components. A water-like bead depicted in light blue that represents the solvent. A gold-like bead that represents the metallic behavior depicted in golden and octadecane molecule composed by six C-type beads that represents surfactants molecules.

The resultant simulations are shown in Fig. **S9** where the left ones **(a)** are obtained if temperature is 343 K. and the left ones **(b)** when the working temperature is equal to 373 K.

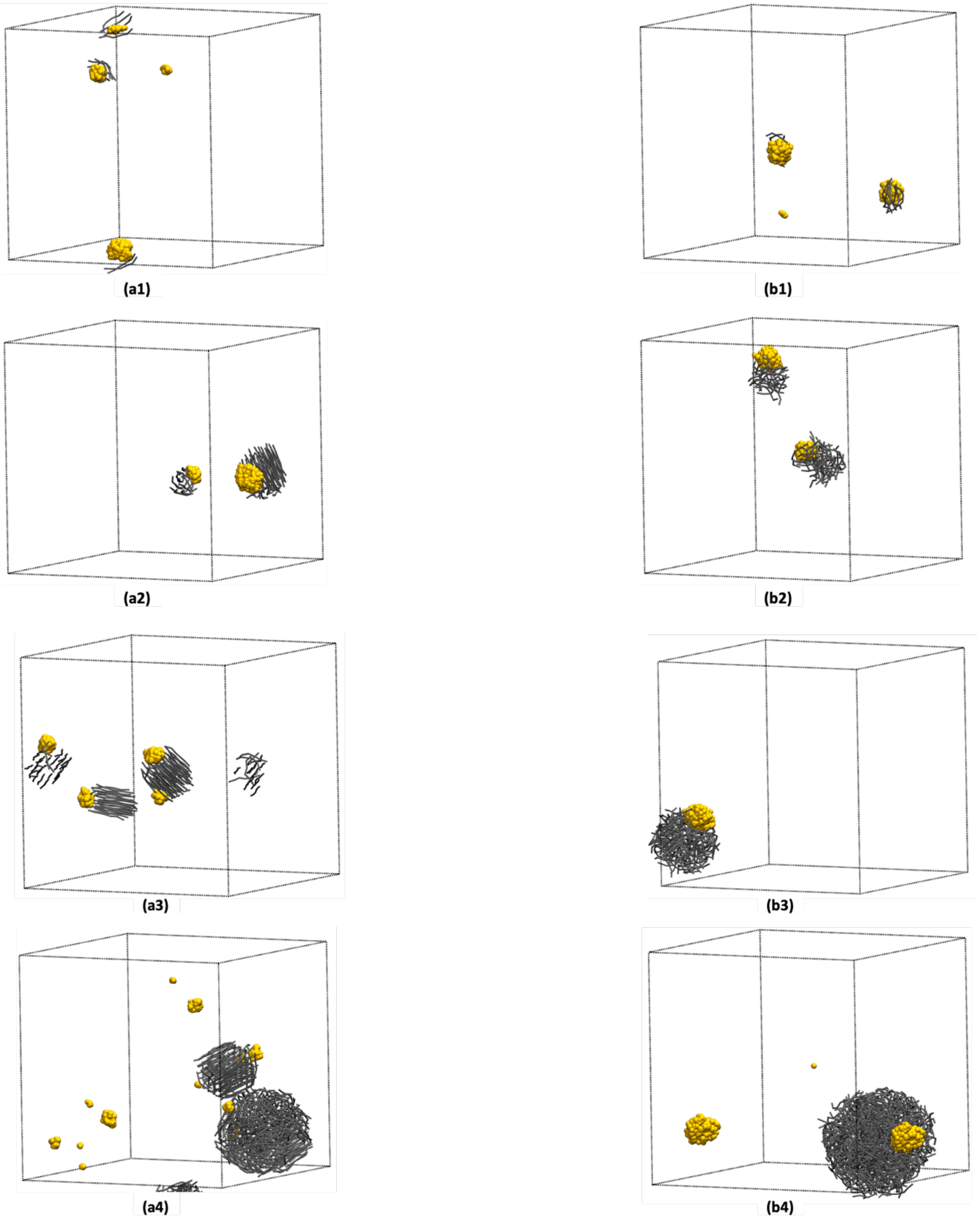

Figure S9- Final structures obtained in 1.35 μs. From 1) to 4) there is represented the last snapshot of four different Au/C-type molar ratios. Images of **(a)** are performed at 343 K of working temperature while images from **(b)** at 373 K.

The total number of Au NPs increase when the number of surfactant molecules is higher when temperatures are lower. On the contrary, when temperature is getting larger the total number of Au NPs is decreased.

Comparing all surfactants. All simulations are performed using 2/1 (Au/surfactant) ratios. All system contains 240,000 water molecules that act as a solvent, 1,000 gold beads and 500 surfactant molecules. Working temperature is equal to 343 K. The total number of Au NPs obtained are shown in figure below (Fig. **S10**).

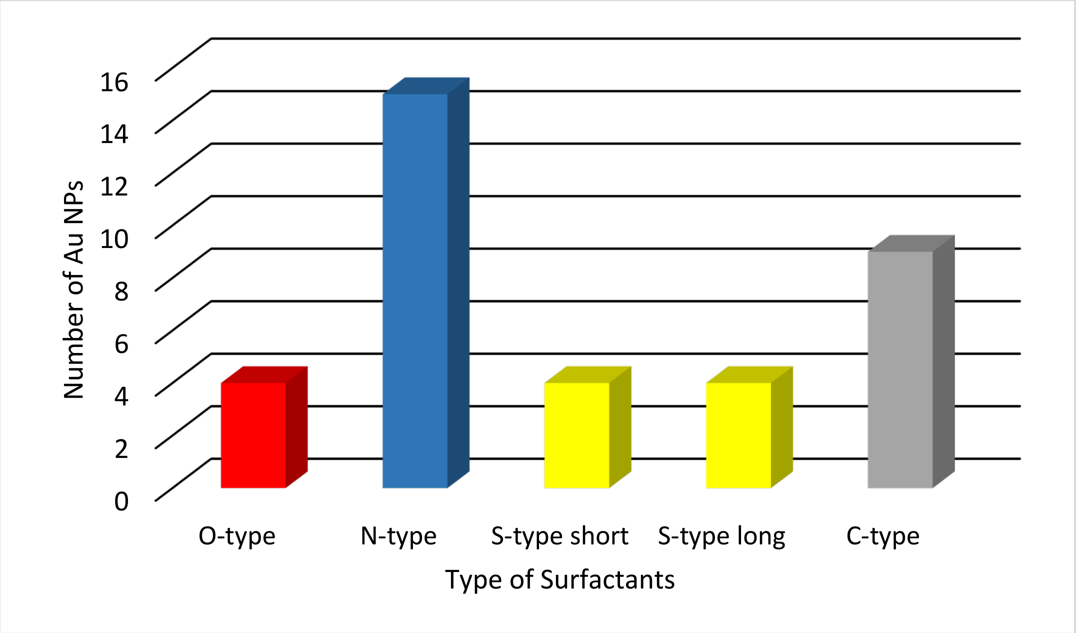

Figure S10- Total number of Au NPs for each surfactant type. Higher number of Au NPs are obtained with larger surfactants (N-type and C-type). The encounter between gold beads is difficulted when hydrocarbon chains are lengthening. Moreover, the number obtain using N-type surfactants is bigger than using C-type surfactants due to the stronger SPR interaction (Au/N=15 and Au/C=55). The main difference between the other surfactants (O-type, S-type short and long) is based on the shape of Au NPs. In case of O-type the Au NPs obtained are spherical while in case of S-type surfactants (short and long) Au NPs adopt a deformed shape. The attraction between thiol groups with gold is higher than the attraction between gold and O-type surfactants (SPR Au/S=5 and SPR Au/O=18). Furthermore, O-type are free in solution while thiol groups are less stable in the solvent (SPR W/O=10 and SPR W/S=54).

The tendency that follows all surfactants according to its concentration and the temperature of the system is collected in Fig. **S11**.

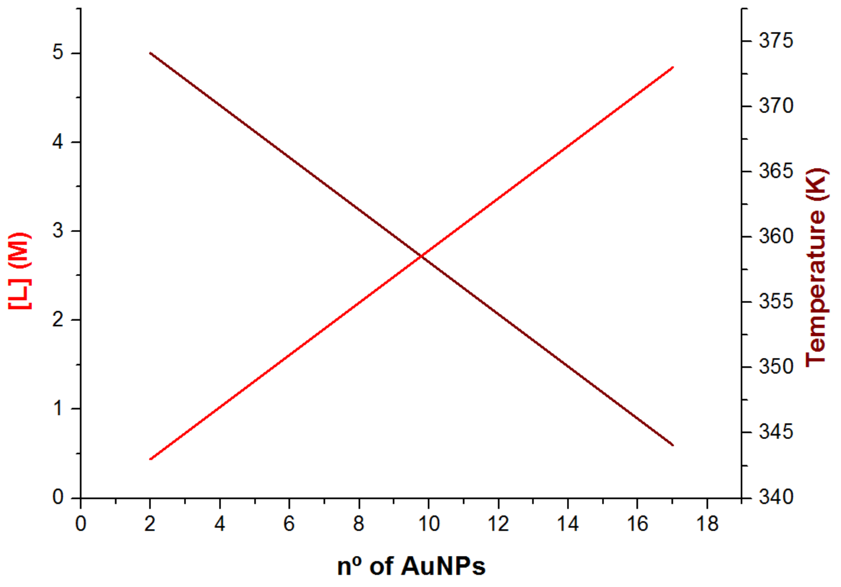

Figure S11- Nº of gold NPs obtained when changing the concentration of surfactants and the working temperature. To obtain Au NPs with higher sizes we need to work with higher temperatures and lower concentration of surfactants. On the other hand, smaller Au NPs are obtained when the temperature is decreased, and the concentration of surfactants is increased.

Methods

DPD formulation

Later, it was slightly modified by Groot and Warren[1,2] to ensure the proper thermal equilibrium state and deriving hydrodynamic variables. The spatial-temporal evolution of each DPD particle is governed by Newton's equations of motion[3]:

$$\frac{d\vec{r_i}}{dt} = \vec{v}_i \frac{d\vec{v}_i}{dt} = \vec{f_i} \tag{1}$$

where, $\vec{r_i}$ is position of particle *i*, $\vec{v}_i$ is the velocity of particle *i*, and $\vec{f_i}$ is the total force acting on bead *i*. For simplicity all masses are normalized to 1. The total force exerted on bead *i* contains three pairwise additive forces:

$$f_i = \sum_{j \neq i} (F_{ij}^C + F_{ij}^D + F_{ij}^R) \tag{2}$$

where F_{ij}^C refers to a conservative force, F_{ij}^D to a dissipative force and F_{ij}^R to a random force. The sum is calculated between all DPD particles within a cut-off radius (R_c) equal to 1. The first

studies the chemical behaviour of the system and is defined by two contributions[1,4]. The first one is defined through equation (3)[5]:

$$F_{ij}^{C} = a_{ij} w^{c}(r_{ij}) \hat{r}_{ij} \tag{3}$$

where a_{ij} is the repulsive force parameter between particles *i* and particle *j*, $\hat{r_{ij}}$ is a unit vector in the direction of r_{ij}, w^{C} is a weight function, and $r_{ij} = r_i - r_j$ V. It derives from a soft potential that tries to capture the effects of the pressure between different particles. The second refers to the elastic contribution, which derives to the system geometry and contains stretching and bending parameters:

$$F_s = -K_r(r_{ij} - r_{eq})\hat{r}_{ij} \tag{4}$$

$$F_\theta = -\nabla\left(\frac{1}{2}K_\theta(\theta - \theta_0)^2\right) \tag{5}$$

Equation (4) describes the harmonic force used to tie two consecutive beads *i* and *j* in a chain and equation (5) is the bond-bending force between consecutive bonds, to control the chain flexibility and strength. Harmonic distance potential parameters (equation (4)) are globally set to r_{eq}= $1d_0$ and K_r= 100($K_B\, T/d_0{}^2$). Parameters for the bond-bending forces (equation (5)) depend on the structure. Values of K_θ= 3 $K_B T/rad^2$ and θ_0=πrad for the linear angles favouring the flexibility of hydrocarbon chains, whereas K_θ= 10 $K_B T/rad^2$ and θ_0=πrad are applied for considering the double bonds. The position of carboxylate groups of citrate is fixed using K_θ= 6 $K_B T/rad^2$ and θ_0=109^{0} maintaining a close-to-center angle.

The other two forces, efficiently act as a thermostat (equation (8)) that keeps the mean temperature of the system constant. F_{ij}^{D} and F_{ij}^{R} have the following forms:

$$F_{ij}^{D} = -\lambda w^{D}(r_{ij})(\hat{r}_{ij} \cdot v_{ij})\hat{r}_{ij} \tag{6}$$

$$F_{ij}^{R} = \sigma w^{R}(r_{ij})\theta_{ij}\hat{r}_{ij} \tag{7}$$

$$w^{D}(r_{ij}) = [w^{R}(r_{ij})]^2 \tag{8}$$

where λ is a friction constant, σ the strength of the random noise, w^{D} and w^{R} are r-dependent weight functions, $v_{ij} = v_i - v_j$ and θ_{ij} is standard random variable of Gaussian distribution[6]. The first, describes the viscous resistance in a real fluid. This force tries to reduce velocity differences between dissipative particles. And the second, is a stochastic force that represents the possible collisions that may occur between atoms within each bead and depends only on the relative positions between particles.

Determination of DPD Interacting Parameters (a_{ij})

To simulate a system, a set of interacting parameters a_{ij}'s between different types of beads must be determined[7] With these non-bonded interactions, DPD simulations should reproduce correct behaviours as observed experimentally. All non-bonded interactions are modelled as soft-repulsive:

$$F_{ij}^{C} = \begin{cases} a_{ij}\left(1-\frac{r_{ij}}{R_c}\right)\hat{r_{ij}}(r_{ij} < R_c) \\ 0(r_{ij} \geq R_c) \end{cases} \tag{9}$$

a_{ij} referred to as *bead-bead repulsion parameters or as DPD soft repulsive interaction parameters* depends on the underlying atomistic parameters[8], and is the repulsive force parameter between particles *i* and particle *j*, $\hat{r_{ij}}$ is a unit vector in the direction of r_{ij}, R_c is the cutoff radius for the repulsive interaction and $r_{ij} = r_i - r_j$ V[3] The strength of the repulsion is set by the value of the repulsion parameter, and it should be $a_{ij} > 0$. The method use for deriving those parameters is based on **Gaussian software package**[9]. Probably the most widely used program package for studying the molecular problems of structure, stability and reaction mechanisms based on quantum chemistry. Although computational quantum studies are primarily concerned by different techniques we are interested on Density functional theory (DFT) calculations. Geometry optimizations are carry out with the Minnesota M06-2X functional, a hybrid meta-GGA in which the energy depends on the occupied orbitals through HF exchange terms and noninteracting kinetic energy densities with LANL2DZ[10]. Polarizable continuum model of water using the integral equation formalism IEFPCM is used.

Binding energies (kcal mol^{-1}) of the system are evaluated by equation (10)[11,12].

$$\Delta E_{binding} = E_{system} - \left(E_{component_1} + E_{component_2}\right) \tag{10}$$

where E_{system} is obtained when W/Au, W/S-type, W/O-type, W/OH-type, W/C-type, W/N-type, Au/Au, Au/S-type, Au/O-type Au/OH-type, Au/C-type, Au/N-type, S/S-type, S/O-type, S-type/OH-type, S-type/C-type, S-type/N-type, O-type/O-type, O-type/OH-type, O-type/C-type, O-type/N-type, OH-type/OH-type, OH-type/C-type, OH-type/N-type, C-type/C-type, C-type/N-type, N-type/N-type dimers are optimized. $E_{component}$ are obtained once single monomers are optimized with the same conditions. All of them are calculated considering a single molecule of water or a single atom of gold and the simplest representation of one bead surfactant. All of them can be exponentially related with a_{ij} (equation (11))

$$a_{ij} = f\left(\Delta E_{binding}\right) \tag{11}$$

Non-bonded interactions are set to mimic that hydrophobic entities repel water, while hydrophilic groups attract water. Therefore, all bead-bead repulsion parameters arise from

water-water interaction (a_{ww}). According to Groot and Warren[1], a_{ww} can be related to experimental thermodynamic properties throughout equation (12) derived from the dimensionless compressibility of water at 300K (κ^{-1}= 16.08):

$$\frac{a_{ww}}{K_B T} = \frac{75}{\rho r_c^3} \tag{12}$$

Where ρ=N/V is the particle number density (V being the volume of the system and N the total number of particles). Thus, ρr_c^3=3 and therefore,

$$\frac{a_{ww}}{K_B T} = 25 \tag{13}$$

Considering that the a_{ww} is equal to 25 and that the binding energy ($\Delta E_{binding}$) resulting from Gaussian is -8.7 kcal mol^{-1}, all the other DPD soft repulsive values are fixed depending on a_{ww} value. All the binding energies that are lower than water binding value will be less repulsive and for this reason the interaction between them will be favourable. On the contrary, the resultant values that are bigger will cause higher values of repulsive parameters and therefore, worst interaction between components.

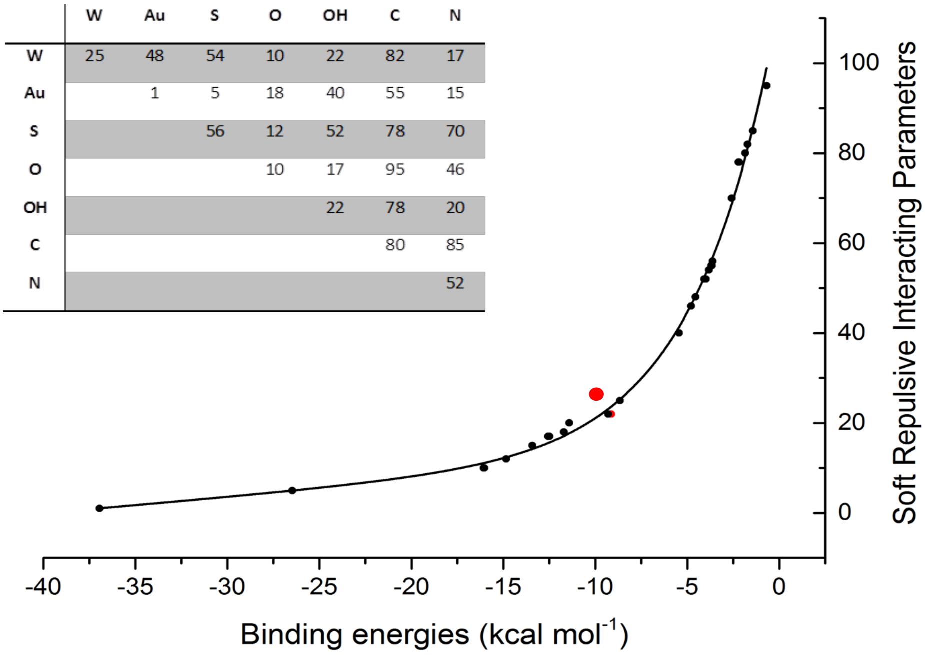

	W	Au	S	O	OH	C	N
W	25	48	54	10	22	82	17
Au		1	5	18	40	55	15
S			56	12	52	78	70
O				10	17	95	46
OH					22	78	20
C						80	85
N							52

Figure S12– Exponential correlation plot between binding energies at LANDL2DZ/M062X level vs. DPD SRPs. Red point represents water-water bead interaction (SPR W/W). Inset: set of resultant SRPs used in this work.

The resultant soft interacting parameters are reasonable considering the fullest chemistry that is behind. Water bead type interacts better with hydrophilic beads (type O) than hydrophobic (type C). The same happen with the other components that can form hydrogen bond making the interaction more stable (for instance O-type/OH-type interaction ($a_{O,OH}$)). O-

type/O-type ($a_{O,O}$) interaction is calculated considering that carboxylic groups are in solution, and therefore, there would be protonated or deprotonated. For this reason, the interaction is considered between one protonated with another deprotonated carboxylic group. Hydrophobic bead type (type C) interact weakly with other components meaning that they are not stable when interact with others. These repulsive parameters follow the tendency explained by Professor Berend Smit[13]. Cross terms (a_{ij}) between gold and other types are measured using the structures commented before except to gold thiol interaction in which, the terminal thiol is deprotonated for representing the strong gold-thiolate bond (Au/S-type; a_{Au,S^-}), similar to gold-gold interaction. In this case, the most attractive interaction is between thiolate and gold (a_{Au,S^-}) beads and the worst one between gold and carbon (Au/C-type; $a_{Au,C}$). Comparing our interacting parameters with Pearson's HSAB theory, we can verify that the growth will be slower if we used thiolate surfactants and faster using hydrocarbon chains.

Simulation conditions. In our simulations we follow Groot and Rabone[14] and set our mass and length reduced units according to the size of three water molecules (except to nucleation procedure). Thus, an standard bead is defined by representing three water molecules ($N_m = 3$). And, therefore, the reduced units of mass are equal to 9E-26 kg. The length scale could be measured by equation (14) considering that water molecules occupied 30Å^3 of volume:

$$d_0^3 = \rho N_m \cdot 30\text{Å}^3 \tag{14}$$

The resultant value is $d_0 = 6.46\text{Å}$. To derive the temperature from reduce to physical units we are based on de Meyer *et al* experiments which estimate the regression parameters for studying the thermodynamic properties of bilayers[13]. As we are interested in providing qualitative structural description of nanometallic particles we choose two different temperatures (low and high) useful for obtaining Au NPs functionalized by citrate surfactants (O-type). According to Turkevich studies about the effect of temperature using sodium citrate solutions we fixed our simulated temperatures (T*) of 0.42 r.u. and 1 r.u. to 343 K and 373 K, respectively[15]. Thus, the relation between simulated and physical units of working temperatures (equation (17)) can be related when solving the following equations:

$$343\text{ K} = \text{a}\,T* + b \qquad where\ T*low = 0.42 \tag{15}$$

$$373\text{ K} = \text{a}\,T* + b \qquad where\ T*high = 1 \tag{16}$$

$$T(°\text{C}) = 51.72\,T* + 48.27 \tag{17}$$

References

[1] Groot, R. D.; Warren, P. B. Dissipative Particle Dynamics: Brinding the gap between atomistic and mesoscopic simulation. *J. Chem. Phys.* 107, 4423–4435 (1997).
[2] Hoogerbrugge, P. J.; Koelman, J. M. V. A. Simulating Microscopic Hydrodynamic Phenomena with Dissipative Particle Dynamics. *Europhys. Lett.* 19, 155–160 (1992).
[3] Juan, S.; Hua, C.; Chen, C.; Sun, X.; Xi, H. Dissipative particle dynamics simulation of a gold nanoparticle system. *Mol. Simul.* 31, 277–282 (2005).
[4] Travis, K. P.; Bankhead, M.; Good, K.; Owens, S. L. New parametrization method for dissipative particle dynamics. *J. Chem. Phys.* 127, 014109 (2007).
[5] Venturoli, M.; Smit, B; Sperotto, M. Simulation Studies of Protein-Induced Bilayer Deformations, and Lipid-Induced Protein Tilting, on a Mesoscopic Model for Lipid Bilayers with Embedded Proteins. *Biophys. J.* 88, 1778-1798 (2005).
[6] Espanol, P.; Warren, P. B. Statistical Mechanics of Dissipative Particle Dynamics. *Europhys. Lett.* 30, 191–196 (1995).
[7] Jacobson, S. H.; Gordon, D. J.; Nelson, G. V.; Balazs, A. Miscible Polymer Blends: Local Interaction Energy Theories and Simulations. *Adv. Mater.* 4, 198–205 (1992).
[8] Maiti, A.; McGrother, S. Bead-bead interaction parameters in dissipative particle dynamics: Relation to bead-size, solubility parameter, and surface tension. *J. Chem. Phys.* 120, 1594–1601 (2004).
[9] Frisch, M. J. *et al.* Gaussian 16, revision C.01; Gaussian, Inc.: Wallingford, CT, 2016.
[10] Zhao Y., Truhlar D. G. Applications and validations of the Minnestoa density functionals. *Chem. Phys. Lett.* 502, 1-13 (2011).
[11] Tardio, S.; Cumpson, P. J. Practical estimation of XPS binding energies using widely available quantum chemistry software. *Surf. Interface Anal.* 50, 5–12 (2018).
[12] Baseden, K. A.; Tye, J. W. Introduction to Density Functional Theory: Calculations by Hand on the Helium Atom. *J. Chem. Educ.* 91, 2116–2123 (2014).
[13] De Meyer, F. J-M.; Venturoli, M.; Smit, B. Molecular simulations of lipid-mediated protein-protein interactions. *Biophys. J.* 95, 1851-1865 (2008).
[14] Groot, R. D.; Rabone, K. L. Mesoscopic Simulation of Cell Membrane Damage, Morphology Change and Rupture by Nonionic Surfactants. *Biophys. J.* 81, 725–736 (2001).
[15] Turkevich, John; Cooper, P.; Hiller, J. A study of the nucleation and growth processes in the synthesis of colloidal gold. *Discuss. Faraday Soc.* 55, 55–75 (1951).
